# Pregnancy and Kidney Diseases: Multidisciplinary Follow-Up and the Vicious Circles Involving Pregnancy and CKD, Preeclampsia, Preterm Delivery and the Kidneys

**DOI:** 10.3390/jcm11092535

**Published:** 2022-04-30

**Authors:** Giorgina Barbara Piccoli, Rossella Attini, Massimo Torreggiani, Gianfranca Cabiddu

**Affiliations:** 1 Néphrologie et Dialyse, Centre Hospitalier Le Mans, 194 Avenue Rubillard, 72037 Le Mans, France; maxtorreggiani@hotmail.com; 2 Department of Obstetrics and Gynecology, Città della Salute e della Scienza, Ospedale Sant’Anna, University of Torino, 10126 Torino, Italy; rossella.attini@gmail.com; 3 Nephrology, Azienda Ospedaliera Brotzu, 09047 Cagliari, Italy; cabiddugianfranca@gmail.com

## 1. Introduction

Thomas Addis, the father of nephrology, once wrote that a clinician is complex, “he is part craftsman, part practical scientist, and part historian” [1]. It is in fact in history that we often find insights that enable us to interpret the times in which we live. Reflecting on the many unsolved issues mentioned in the previous editorial [2], we would like to draw the reader’s attention to the circular nature of the relationship between kidney and pregnancy and to two vicious circles, the focus of two extraordinary papers, one published at the beginning and one at the end of the 20th century [3,4].

## 2. From CKD to Preeclampsia and Back

**Figure 1:**
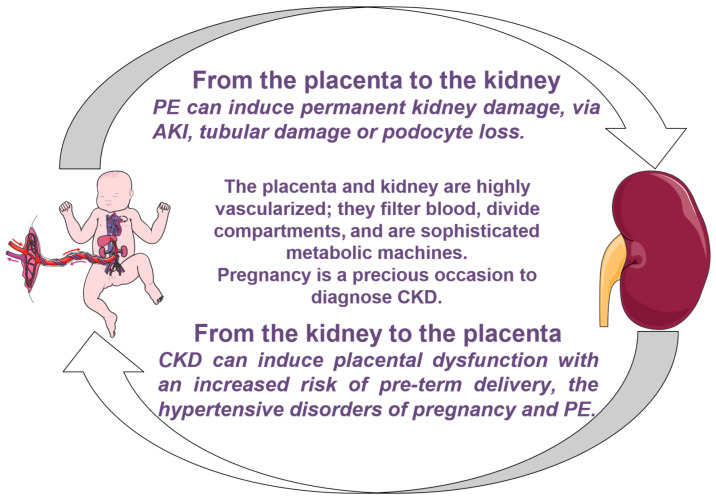
The vicious circle from the placenta to the kidney and from the kidney to the placenta. Adapted from [5]. PE: preeclampsia, AKI: acute kidney injury, CKD: chronic kidney disease.

**Figure 2:**
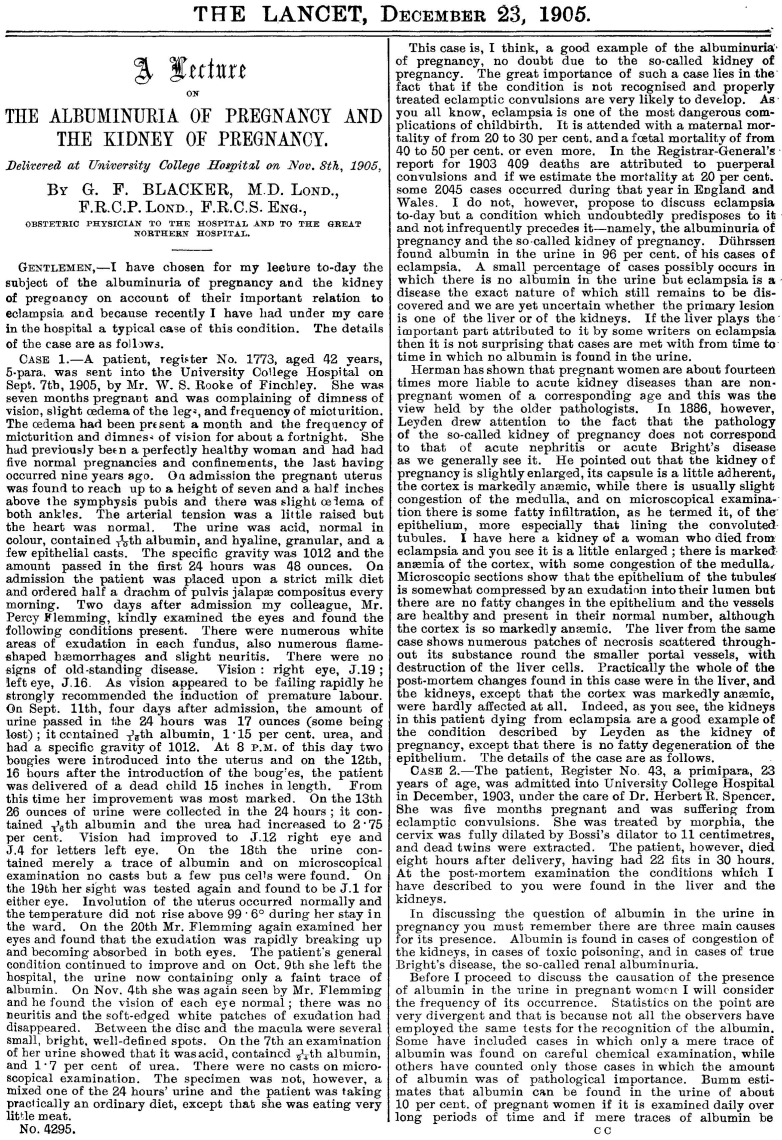
The front page of the paper “The albuminuria of pregnancy and the kidney of pregnancy”, *The Lancet*, 23 December 1905 [3].

Pregnancy complications affect the kidney and kidney diseases affect pregnancy complications (Figure 1). The lecture entitled “The albuminuria of pregnancy and the kidney of pregnancy”, which was published in The Lancet on 23 December 1905 [3] (Figure 2) deals with five emblematic cases. The first was a 42-year-old woman, at her sixth pregnancy, who complained of mild visual blurring and oedema in her seventh month of gestation; mild hypertension was found, and after a phase of oliguria/anuria, with increased proteinuria, symptoms disappeared with the delivery of a child dead in utero. The second case, a primiparous, 23-year-old woman, with eclampsia at five months of gestation, died after the delivery of twins, dead in utero. This case allows the author to discuss the differential diagnosis, which he analyses as follows: “When you discover albumin in the urine of a pregnant woman you must bear in mind that it may be due to three very distinct conditions. The patient may be the subject of acute nephritis or acute Bright’s disease; she may be suffering from chronic nephritis aggravated by the pregnancy, or she may be suffering from the albuminuria of pregnancy and the so-called kidney of pregnancy which [omissis] does not correspond quite to any of the varieties of acute nephritis or acute Bright’s disease” [3]. In the third case, a young woman, with a history highly suggestive of Bright’s disease, the “toxemic theory” supported the idea that albuminuria is caused by the action of “certain toxins” circulating in the mother’s blood [3]. The woman died of uraemia after delivering a child in the eighth month of pregnancy. In the fourth case, with a similarly grim prognosis, the woman had a small shrunken kidney, probably from “chronic pyelonephritis”. The questions the author posed still hold true: “What dangers does the kidney of pregnancy expose the patient to?” His answer is that “They are mainly three in number: first of all there is eclampsia, which occurs in about one in every five cases of the kidney of pregnancy; secondly, there is the subsequent development of chronic nephritis; and thirdly, the danger of partial or complete loss of vision due to the changes in the eye. Another danger that will occur to you is that of uraemia but when this takes place the case is more likely to be one of acute nephritis or acute Bright’s disease than of the kidney of pregnancy” [3].

Further on, the author considers the long-term dangers, and the risks of recurrence of the “kidney of pregnancy”, stating that this is presumably higher in cases that occur early during gestation. Likewise, the higher risks of adverse pregnancy outcomes in women with Bright’s disease, which, at the time, encompassed all chronic diseases of the kidneys, in the absence of imaging and well before kidney biopsy became available, were underlined, together with the difficulty in discriminating during, and often even after, pregnancy between “kidney of pregnancy” and Bright’s disease. 

One hundred years later, we know more about this first vicious circle (Figure 1) from the diseased kidney to the placenta and from the placenta to the diseased kidney. Recent studies highlight the fact that the circulating biomarkers recognized by Blacker have distinct behaviours in preeclampsia, chronic kidney disease (CKD) and superimposed pre-eclampsia [6,7]. Furthermore, all forms of early CKD are now acknowledged to be associated with higher risks of adverse pregnancy outcomes, and this holds true even for “trivial” conditions, such as a history of nephrolithiasis, previous acute kidney injury (AKI) or Stage 1 CKD [8,9]. With this in mind, several groups, including ours, strongly advocate that serum creatinine be included among the tests routinely prescribed at the start of pregnancy or in pre-gestational assessment [10]. If we knew more about the effects even initial CKD or a “healthy” reduction in kidney tissue (e.g., kidney donation), have on a subsequent pregnancy, we could obtain additional information on the detrimental association between preeclampsia, other hypertensive disorders of pregnancy and future maternal cardiovascular and kidney health [11]. 

We still need, however, to cast light on the effect of the different kidney diseases on pregnancy, to try to better understand whether quantity or quality of tissue counts, and to determine what effect specific diseases have on pregnancy outcomes. Is the relationship between the hypertensive disorders of pregnancy and subsequent kidney health an effect of hypertensive and proteinuria insult to kidney tissue? Is this effect mediated by loss of podocytes, or is it the reflection of a pre-existent kidney disease, now found in at least 20% of cases when searched for, or is it the first sign of a subclinical kidney injury, for example in the case of obesity [8]? We hope that some of these questions will be answered in the present issue. 

## 3. Being Born Small and the Risk of Having Small Babies

The second paper that we would like to comment on appeared in Epidemiology in 1999 [4]. Written nearly a decade before the pivotal paper by Vikse and his colleagues was published [12], its title not only highlights the importance of preeclampsia in the future development of CKD, but also demonstrates awareness of the second kidney-related vicious circle in pregnancy: small, or preterm babies, who have, in turn, a higher risk of complicated pregnancies, and of giving birth to small babies. 

According to this study, being born small, and as we now better acknowledge, small for gestational age, increases by 4 to 6 times the risk of having a complicated pregnancy, leading in turn to an increased risk of giving birth to a “small baby” [4]. 

**Figure 3:**
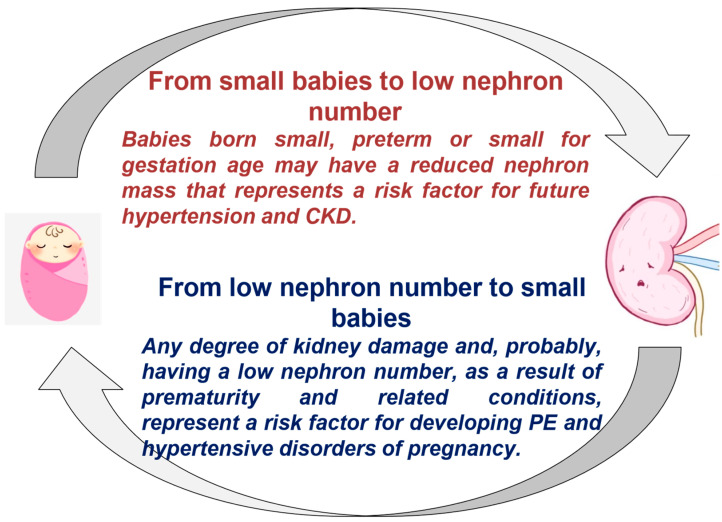
The vicious circle from small baby to complicated pregnancy and back to small baby. PE: preeclampsia, CKD: chronic kidney disease.

In more recent studies, being born small for gestational age has increasingly been associated with the development of hypertension, metabolic syndrome and kidney disease in adulthood [13,14,15]. Indeed, we now know that the slow, and sometimes unpredictable, maturation of the kidney tissue is probably one of the reasons for this increased risk, and may also be the mediator of the increased risk of the hypertensive disorders of pregnancy observed in the pregnancies of women born small, preterm, or small for gestational age [14] (Figure 3). 

Once more, even though our knowledge of these interrelated events has increased enormously in recent years, being born small (in all its variants) is not considered a significant risk factor for the development of the hypertensive disorders of pregnancy, or included in counselling. The vast and fascinating field of epigenetics is open for discussion, while, possibly because of the heterogeneity of the hypertensive disorders of pregnancy, what constitutes a favouring genetic background remains unknown. 

While shedding light on these and other open issues, including parenthood, is quite an ambitious task, we hope that our series will contribute to the field, adding one more drop to the ocean and creating a butterfly effect.

## References

[1] Addis T. (1948). Glomerular Nephritis, Diagnosis and Treatment.

[2] Piccoli G.B., Attini R., Cabiddu G. (2018). Kidney Diseases and Pregnancy: A Multidisciplinary Approach for Improving Care by Involving Nephrology, Obstetrics, Neonatology, Urology, Diabetology, Bioethics, and Internal Medicine. J. Clin. Med..

[3] Blacker G.F. (1905). A Lecture on the albuminuria of pregnancy and the kidney of pregnancy. Lancet.

[4] Innes K.E., Marshall J.A., Byers T.E., Calonge N. (1999). A woman’s own birth weight and gestational age predict her later risk of developing preeclampsia, a precursor of chronic disease. Epidemiology.

[5] Piccoli G.B., Alrukhaimi M., Liu Z.-H., Zakharova E., Levin A., Li P.K.T., Garcia-Garcia G., Benghanem-Gharbi M., Kalantar-Zadeh K., Kernahan C. (2018). What we do and do not know about women and kidney diseases; questions unanswered and answers unquestioned: Reflection on World Kidney Day and International Women’s Day. J. Nephrol..

[6] Zeisler H., Llurba E., Chantraine F., Vatish M., Staff A.C., Sennstrom M., Olovsson M., Brennecke S.P., Stepan H., Allegranza D. (2016). Predictive Value of the sFlt-1:PlGF Ratio in Women with Suspected Preeclampsia. N. Engl. J. Med..

[7] Rolfo A., Attini R., Tavassoli E., Neve F.V., Nigra M., Cicilano M., Nuzzo A.M., Giuffrida D., Biolcati M., Nichelatti M. (2015). Is It Possible to Differentiate Chronic Kidney Disease and Preeclampsia by means of New and Old Biomarkers? A Prospective Study. Dis. Markers.

[8] Cabiddu G., Mannucci C., Fois A., Maxia S., Chatrenet A., Osadolor S., Kimani E., Torreggiani M., Attini R., Masturzo B. (2021). Preeclampsia is a valuable opportunity to diagnose chronic kidney disease: A multicentre study. Nephrol. Dial. Transplant..

[9] Tangren J.S., Wan Md Adnan W.A.H., Powe C.E., Ecker J., Bramham K., Hladunewich M.A., Ankers E., Karumanchi S.A., Thadhani R. (2018). Risk of Preeclampsia and Pregnancy Complications in Women With a History of Acute Kidney Injury. Hypertension.

[10] Piccoli G.B., Chatrenet A., Cataldo M., Torreggiani M., Attini R., Masturzo B., Cabiddu G., Versino E., Kidney and Pregnancy Study Group of the Italian Society of Nephrology (2022). Adding creatinine to routine pregnancy tests: A decision tree for calculating the cost of identifying patients with CKD in pregnancy. Nephrol. Dial. Transplant..

[11] Garg A.X., Nevis I.F., McArthur E., Sontrop J.M., Koval J.J., Lam N.N., Hildebrand A.M., Reese P.P., Storsley L., Gill J.S. (2015). Gestational hypertension and preeclampsia in living kidney donors. N. Engl. J. Med..

[12] Vikse B.E., Irgens L.M., Leivestad T., Skjaerven R., Iversen B.M. (2008). Preeclampsia and the risk of end-stage renal disease. N. Engl. J. Med..

[13] Brenner B.M., Garcia D.L., Anderson S. (1988). Glomeruli and blood pressure. Less of one, more the other?. Am. J. Hypertens..

[14] Luyckx V.A., Brenner B.M. (2020). Clinical consequences of developmental programming of low nephron number. Anat. Rec..

[15] Mericq V., Martinez-Aguayo A., Uauy R., Iniguez G., Van der Steen M., Hokken-Koelega A. (2017). Long-term metabolic risk among children born premature or small for gestational age. Nat. Rev. Endocrinol..

